# Th17 Cells in Depression: Are They Crucial for the Antidepressant Effect of Ketamine?

**DOI:** 10.3389/fphar.2021.649144

**Published:** 2021-04-15

**Authors:** Meiying Cui, Wanlin Dai, Jing Kong, Hongzhi Chen

**Affiliations:** ^1^ Department of Anesthesiology, Shengjing Hospital, China Medical University, Shenyang, China; ^2^ Innovation Institute of China Medical University, Shenyang, China; ^3^ Department of General Surgery, Shengjing Hospital of China Medical University, Shenyang, China

**Keywords:** ketamine, th17 cell, depression, immune, cytokine

## Abstract

**Background:** Major depressive disorder is associated with inflammation and immune processes. Depressive symptoms correlate with inflammatory markers and alterations in the immune system including cytokine levels and immune cell function. Th17 cells are a T cell subset which exerts proinflammatory effects. Th17 cell accumulation and Th17/Treg imbalances have been reported to be critical in the pathophysiology of major depressive disorder and depressive-like behaviors in animal models. Th17 cells are thought to interfere with glutamate signaling, dopamine production, and other immune processes. Ketamine is a newly characterized antidepressant medication which has proved to be effective in rapidly reducing depressive symptoms. However, the mechanisms behind these antidepressant effects have not been fully elucidated.

**Method:** Literature about Th17 cells and their role in depression and the antidepressant effect of ketamine are reviewed, with the possible interaction networks discussed.

**Result:** The immune-modulating role of Th17 cells may participate in the antidepressant effect of ketamine.

**Conclusion:** As Th17 cells play multiple roles in depression, it is important to explore the mechanisms of action of ketamine on Th17 cells and Th17/Treg cell balance. This provides new perspectives for strengthening the antidepressant effect of ketamine while reducing its side effects and adverse reactions.

## Introduction

**FIGURE 1:**
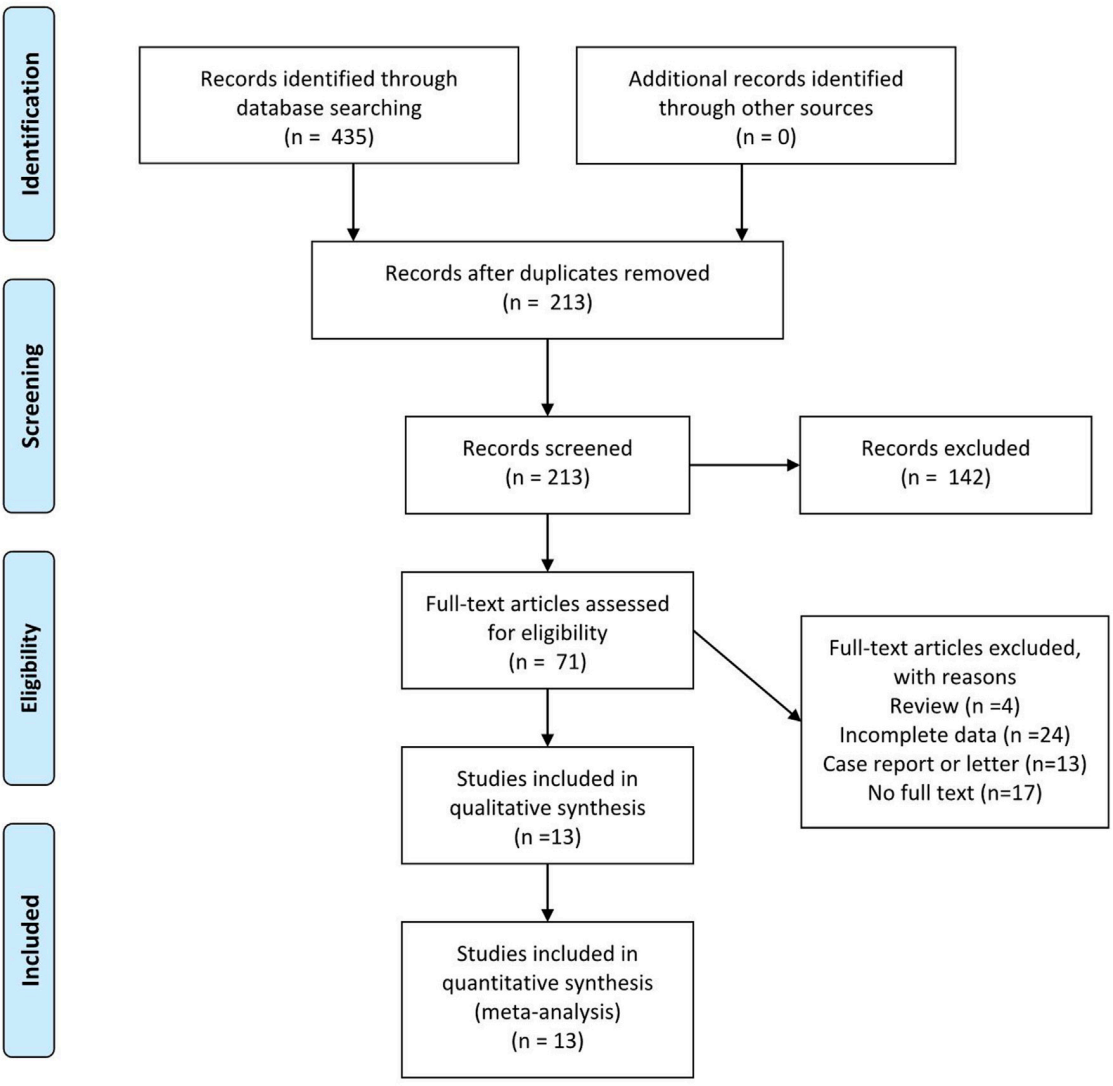
Preferred reporting items for systematic review and meta-analysis (PRISMA) flow diagram.

Th17 cells are a group of proinflammatory T helper cells which play important roles in various inflammatory and autoimmune diseases such as rheumatoid arthritis, multiple sclerosis, psoriasis, and inflammatory bowel disease (Yang et al., 2014). The major cytokines secreted by Th17 cells are IL-17, IL-21, and IL-22, with IL-17 as its most important functional cytokine (Korn et al., 2009). The IL-17 family consists of six members (IL-17A-F). IL-17A, which is produced by activated T cells, and stimulates and regulates immunity (Kolls and Lindén, 2004). IL-17 receptors are divided into three subtypes, IL-17RA, IL-17RB, and IL-17RC, with different combinations exerting different functions (Gaffen, 2009). Th17 cells are mainly derived from CD4^+^ naive T cells (Korn et al., 2009) and are normally induced by a combination of TGF-β and IL-6 (McGeachy and Cua, 2008), under regulation by the transcription factors RORγt and STAT3 (Korn et al., 2009). Th17 differentiation can also be induced by combined stimulation by IL-1β, IL-23, and IL-6 (Revu et al., 2018). Besides the proinflammatory characteristics of Th17 cells, a new subpopulation, Treg17 cells, which shows anti-inflammatory features, has been recently reported. Their major functioning cytokine is IL-10 (Kluger et al., 2016). A detailed literature search was performed using depression, ketamine, and Th17 as keywords in the Web of Science, Cochrane, PubMed, and CNKI online databases (last search date: December. 30, 2020) without region, publication type, or language restrictions. The search strategy applied to PubMed is listed below: ((((depression) OR (depressive symptoms)) OR (major depression disorder)) AND (((Th17 cell) OR (T helper 17 cell)))) OR (((th17 cell) OR (t helper 17 cell)) AND ((ketamine) OR (esketamine))). Figure 1 illustrates the PRISMA flow chart of literature search strategies.

### Th17 Cells in CNS

Th17 cells have strong plasticity, and both pathogenic and nonpathogenic Th17 cell subsets have been described in past reports (Lee et al., 2012). Under physiological conditions, the number of Th17 cells is very small, almost undetectable in peripheral blood (Shen et al., 2009). Th17 cells are mainly distributed in the intestinal mucosa and protect against extracellular pathogens such as *Staphylococcus aureus* and *Klebsiella spp*. by secreting IL-17A, IL-17F, IL-21, and IL-22 (Korn et al., 2009). In addition to functioning as adaptive immune cells in defending against pathogen invasion, Th17 cells and their major effector cytokine IL-17 play important roles in memory, social behavior, and structural brain development (Derecki et al., 2010). Many studies have confirmed the homeostatic function of T cells in the central nervous system (CNS), including their role in hippocampal-dependent learning (Kipnis et al., 2004; Ziv et al., 2006; Brynskikh et al., 2008; Wolf et al., 2009; Wolf et al., 2009; Kipnis et al., 2012), stress responses (Cohen et al., 2006; Lewitus and Schwartz, 2009) and their protective effects against neurodegeneration and CNS injury (Moalem et al., 1999; Kipnis et al., 2002; Serpe et al., 2003; Frenkel et al., 2005). The function and development of Th17 cells are closely related to the function and development of Treg cells, and the balance of the two cell types is critical to immune homeostasis (Park and Pan, 2015; Diller et al., 2016). Under physiological or non-autoimmune conditions, Th17 cells are not destructive to the brain but are the key brain nutrient cells that regulate mood and hippocampal growth. Transferring T cells to animals with T cell defects can restore normal behavior and reverse impaired learning ability, hippocampal neurogenesis, and brain growth factor expression (Ziv and Schwartz, 2008; Wolf et al., 2009). Specifically, a study by Kemperman et al. showed that adoptive transfer of Th17 cells rich in myelin-reactive T cell receptors can ameliorate compromised hippocampal neurogenesis and thus further corroborated the protective effect of Th17 cells on brain function (Niebling et al., 2014). However, whether these passively infused Th17 cells migrate to the CNS or secrete effector cytokines such as IL-17 to affect the CNS remotely is still unclear. In addition, Th17 cells may also play an anabolic role in maintaining the function and structural integrity of the brain. In healthy people, Th17 cells are positively correlated with greater white matter integrity. A study by Poletti et al*.* showed that under physiological conditions, the percentage of Th17 cells in circulation is positively correlated with higher fractional anisotropy of fiber bundles related to functional integrity in the brain (Poletti et al., 2017).

Conversely, Th17 cells also play important roles in immunopathology in the CNS. The number of Th17 cells and expression of IL-17 in CNS increase under pathological conditions such as multiple sclerosis, resulting in neuroinflammation and neurotoxicity (Sie et al., 2014). Under pathological conditions, the source of IL-17 is not restricted to Th17 cells; IL-17 is also secreted by astrocytes, microglia, and neurons (Moynes et al., 2014). The IL-17 receptors, IL-17RA, and IL-17RC are expressed by astrocytes, microglia, and endothelial cells, and these cells become activated upon IL-17 binding to its receptors (Zimmermann et al., 2013; Sun et al., 2017), resulting in release of NO and iNOS (Kawanokuchi et al., 2008) and neurotoxicity. The pathogenic Th17 cell subpopulations are considered to possess different characteristics from traditional Th17 cells. For example, in experimental autoimmune encephalitis, pathogenic Th17 cells are characterized by secretion of IL-17 and IFN-γ (Yasuda et al., 2019). The pathogenicity of Th17 cells is also associated with increased expression of CCR6 (Chen et al., 2017), IL-23R (Lee et al., 2017), CD5L (Davis et al., 2015), and CXCR5 (Beurel et al., 2013). This pathogenicity is also determined by localization; for instance, in the CNS, Th17 cells expressing T-bet are pathogenic, while in the intestine, Th17 cells not expressing T-bet are pathogenic (Krausgruber et al., 2016). Moreover, microRNA and RNA-binding proteins are also important regulators of Th17 cell pathogenicity (Ichiyama et al., 2016). In addition, Th17 cells can contribute to blood-brain barrier destruction, increasing its permeability, thus facilitating other immune cells entering the CNS. For example, IL-17A promotes monocyte migration across the blood-brain barrier by upregulating intracellular adhesion molecule 1 (Huppert et al., 2010).

### Th17 Cells and Depression

Th17 cells are suggested to play important roles in the pathophysiology of depression. The number of Th17 cells and the expression of IL-17A and RORγt are upregulated in peripheral blood from patients with major depressive disorder (MDD) (Chen et al., 2011; Davami et al., 2016). However, these results only suggest a possible correlation between Th17 cells and depression but are inadequate to establish a causal link between them. It is possible that Th17 cells under pathological conditions show pathogenic characteristics. Studies by Beurel et al. and Nadeem et al. showed that infusion of Th17 cells or IL-17 induced a depression-like behavior in the CRS (chronic restraint stress) mouse model (Beurel et al., 2013; Nadeem et al., 2017). The percentage of Th17 cells in the brains of CRS mice is three-fold higher than that of control mice. Moreover, RORγt knockout or IL-17A neutralizing antibody administration resulted in resistance to learned helpless in mice (Beurel et al., 2013). Similarly, IL-17A antibody treatment in psoriatic patients with moderate to severe depression resulted in a significant reduction in depressive symptoms in 40% of the patients (Griffiths et al., 2017). Therefore, Th17 cells and IL-17A are considered potential therapeutic targets for depression.

Furthermore, Liu et al. found that IL-17 knockout significantly improved neurogenesis (Liu et al., 2014). The study by Beurel et al*.* also found that adoptively transferring Th17 cells to *Rag2* ^−/−^ mice devoid of endogenous T cells can increase their susceptibility to learned helplessness. Transfer of Th17 cells into wild-type mice leads to accumulation in the hippocampus and induces Th17 cells to differentiate *in situ*. Th17 cells in the hippocampus of mice with learned helplessness express the surface markers CCR6 and IL-23R, characteristic of pathological Th17 cells, and the markers CXCR5 and PD-1, typical of follicular cells. Knockout of CCR6 in Th17 cells blocked their ability to promote learned helplessness (Beurel et al., 2018). This evidence further supports that Th17 cells that contribute to depression are a subset of pathological Th17 cells. Since anxiety and pain are important inducing factors of depressive symptoms, it is possible that Th17 cells exert their effects in depression by increasing anxiety or reducing pain threshold. However, infusion of Th17 cells did not alter anxiety or pain sensitivity in animal models. The Th17/Treg balance is a key regulator of immune status, and an imbalance between them has been reported in patients with depression (Hong et al., 2013; Waisman et al., 2015). Increases in Th17 cell numbers and decreases in Treg cell numbers are most frequently reported, but simultaneous decreases have also been reported (Grosse et al., 2016). In contrast, decreases in Th17 cells and increases in Tregs are seen in a chronic unpredictable mild stress model of mice (Hong et al., 2013), which is postulated to be associated with transformation of Th17 cells into Tregs. A transition to Tregs is a final destination of most effector T cells and is not unique to Th17 cells.

There are several studies showing that IL-17A levels are not associated with depressive symptoms (Liu et al., 2012; Kim et al., 2013; Spanemberg et al., 2014). For example, Saraykar hypothesized that there is no correlation between IL-17A and depression in late life, but there is a possible association between IL-17A and cognitive dysfunction (Saraykar et al., 2018). This could suggest that Th17 cells may exert their effects in depression via cytokines other than IL-17, such as IL-21 and IL-22. However, a study by Davami et al. reported that the peripheral IL-21 levels are not significantly different between the patients with depression and healthy controls (Davami et al., 2016). Another possible explanation is that IL-17A expression could vary during disease progression or among depression subtypes. Other confounding factors such as body mass index and smoking may also bias the correlations. Gender differences are another possible interfering factor because most animal models only include males. Notably, a protective role of Th17 cells in depression is also suggested by some reports. In Niebling’s study, infusion of Th17 cells restored neurogenesis in the hippocampus (Niebling et al., 2014). In another study by Tfilin, IL-17 infusion regulated hippocampal neurogenesis as well as spatial learning in a mouse model (Tfilin and Turgeman, 2019). Moreover, an increase in the Th17/Treg ratio is beneficial for white matter integrity in patients with bipolar disorder (Poletti et al., 2017).

The exact mechanisms of Th17 cell action in the pathology of depression are still unclear. Recent ideas include neuroinflammation, perturbation of dopamine production, IL-17A-mediated glutamate excitotoxicity (Kostic et al., 2017), promotion of neuroprogression (Swardfager et al., 2014), and inhibition of hippocampal neurogenesis by IL-17 (Liu et al., 2014). Th17 cells are mainly found in the intestinal mucosa and are thought to enter peripheral blood under pathological circumstances. Intestinal immune cells communicate with the CNS via the gut-brain axis, including translocation of immune cells themselves or secretion of cytokines into systemic circulation to then enter the brain (Powell and MacDonald, 2017). In germ-free mice, there is impaired production of Th17 cell differentiation factors IL-6 and IL-1β, including alleviation of depressive symptoms (Fung et al., 2017), suggesting an important role of intestinal gut microbiota-immune system-CNS interactions in the pathophysiology in depression. Thus, the development of depressive symptoms possibly results from altered Th17 cell numbers and function and stress-induced gut microbiota changes.

### Antidepressant Effects of Ketamine

The rapid and sustained antidepressant effects of ketamine are the subject of many current studies (Murrough et al., 2017). A single dose of ketamine induces a rapid and sustained antidepressant effect (Corriger and Pickering, 2019), but the underlying mechanisms remain unclear. Recent studies hypothesize NMDA receptor inhibition (Murrough et al., 2017), AMPA receptor activation (Freudenberg et al., 2015), mTOR signaling pathway activation, increased expression of synaptic proteins, regulation of neuroinflammation (Yang et al., 2020), modulation of calcium channels and the Kir4.1 potassium channel in the lateral habenula (Cui et al., 2018; Yang et al., 2018), and regulation of neuronal resting potential as potential mechanisms. Based on the crucial role of Th17 cells and IL-17 in depression, it can be postulated that ketamine’s antidepressant effects may be associated with Th17 cells and IL-17. However, since the alteration of cytokine expression and function are not immediate, it is possible that ketamine exerts its rapid antidepressant effect via regulating ion channels within a short period of time, and the sustained antidepressant effects are associated with modulating cytokine networks in a slow and continuous manner.

### Effects of Ketamine on Th17 Cells

**FIGURE 2:**
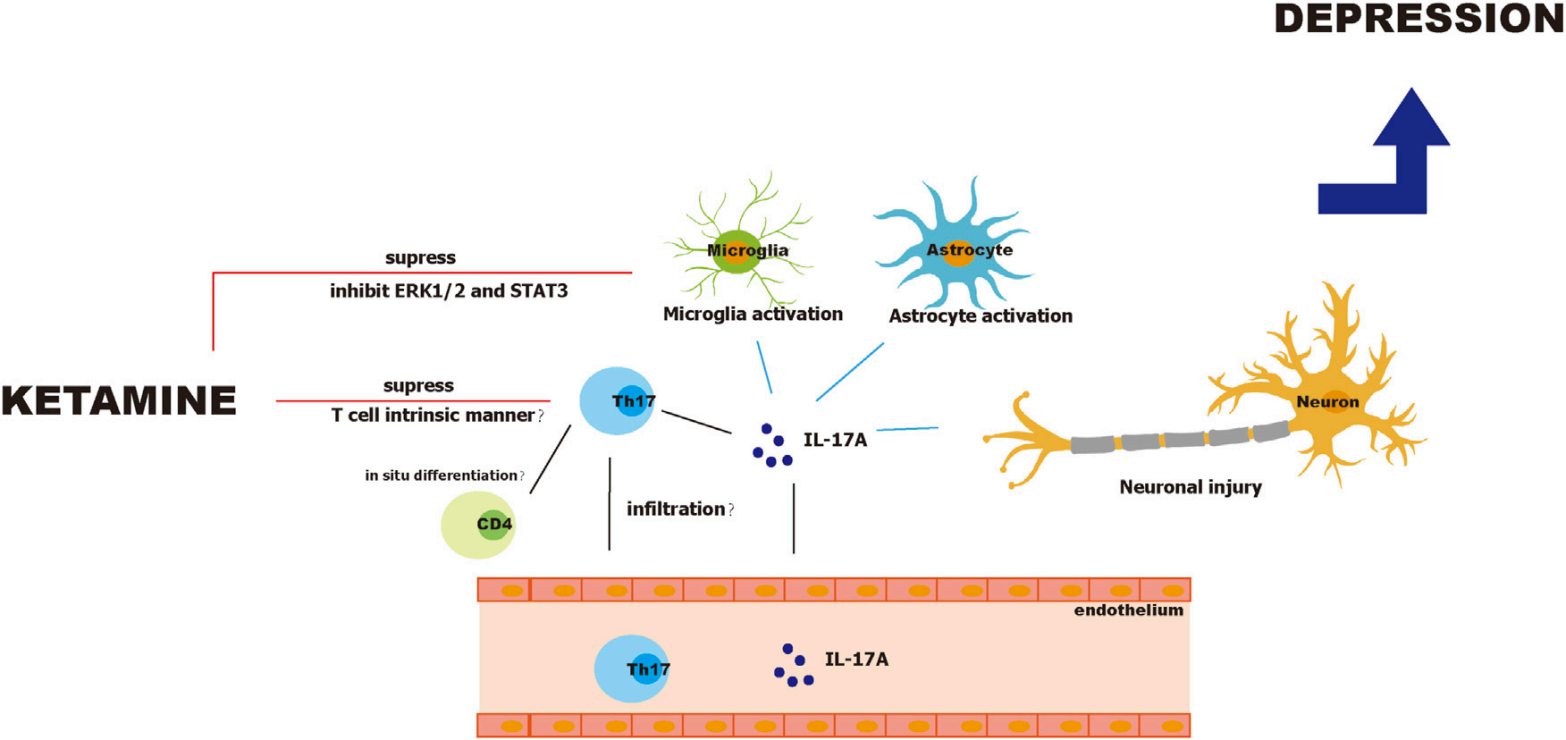
Pathophysiology of Th17 cells in depression and antidepressant effects of ketamine.

Ketamine suppresses differentiation and proliferation of Th17 cells in a mouse model of experimental autoimmune encephalitis (Lee et al., 2017). Further, Lee demonstrated that ketamine inhibited Th17 cell differentiation in a T cell intrinsic manner, because ketamine did not affect the secretion of Th17 cell differentiation factors such as IL-1β, IL-6, and IL-23 by dendritic cells. In addition, ketamine did not induce apoptosis of Th17 cells. However, the study by Lee did not exclude other sources of IL-17 such as microglia, astrocytes, and neurons. Notably, ketamine inhibits LPS-induced activation of microglia through ERK1/2 inhibition (Chang et al., 2009) and regulates the STAT3 pathway in microglia (Ho et al., 2019). Interestingly, ketamine also specifically inhibits pathogenic Th17 cells. Lee reported that ketamine reduces numbers of IFN-γ^+^ IL-17^+^, and GM-CSF^+^IL-17^+^ T cells but not the total number of IL-17^+^ T cells (Lee et al., 2017). Moreover, a study by Ghosh et al*.* found that IFN-γ^+^ IL-17^+^ T cells in patients with MDD secrete more IL-17, and their intracellular levels of IL-17 significantly correlate with the HDRS score (Ghosh et al., 2020). This suggests that ketamine may suppress pathogenic Th17 cell subsets but not the whole Th17 cell population. In contrast, in ketamine cystitis where ketamine overuse causes inflammation in the bladder, the number of Th17 cells is reportedly increased (Fan et al., 2017). Since ketamine cystitis is mostly seen in chronic ketamine users and ketamine abusers, it is possible that ketamine administration in a short period and safe dosage reduces the number of pathogenic Th17 cells, while in chronic and unguided users, Th17 cells are increased, resulting in local chronic inflammation and unexpected side effects. A safe dosage window and interval are required for ketamine’s antidepressant effects; it is therefore possible that ketamine exerts beneficial effects against depression by regulating Th17 cells when used within the appropriate dosage and intervals but causes adverse effect such as ketamine cystitis, psychotomimetic effects (Zanos et al., 2018), and cognitive dysfunction (Ke et al., 2018; Ide et al., 2019) under improper application. Figure 2 illustrates the pathophysiology of Th17 cells during depression and ketamine’s antidepressant effects.

## Conclusion

Increasing evidence suggests that inflammation and the adaptive immune system play important roles in the progression of MDD. Th17 cells are suggested to have crucial impact on the pathogenesis of depression by promoting CNS neuroinflammation and inducing neurotoxicity. The imbalance of Th17/Treg cells is reportedly involved in the progression of depression. Recently, ketamine has been used to quickly relieve depressive symptoms and suicidal ideation in patients with depression. Despite its significant beneficial effects, its clinical safety is still unclear. The mechanisms of ketamine’s antidepressant effects involve a wide range of biological processes. As Th17 cells play multiple roles in depression, it is important to explore the actions of ketamine on Th17 cells and Th17/Treg balance. This would provide new perspectives for understanding the antidepressant effects of ketamine and reducing its adverse side effects.

## References

[B1] Beurel E. Harrington L. E. Jope R. S. (2013). Inflammatory T helper 17 cells promote depression-like behavior in mice. Biol. Psychiatry 73 (7), 622–630. 10.1016/j.biopsych.2012.09.021 23174342 PMC3582833

[B2] Beurel E. Lowell J. A. Jope R. S. (2018). Distinct characteristics of hippocampal pathogenic TH17 cells in a mouse model of depression. Brain Behav. Immun. 73, 180–191. 10.1016/j.bbi.2018.04.012 29698707 PMC6287768

[B3] Brynskikh A. Warren T. Zhu J. Kipnis J. (2008). Adaptive immunity affects learning behavior in mice. Brain Behav. Immun. 22 (6), 861–869. 10.1016/j.bbi.2007.12.008 18249087

[B4] Chang Y. Lee J. J. Hsieh C. Y. Hsiao G. Chou D. S. Sheu J. R. (2009). Inhibitory effects of ketamine on lipopolysaccharide-induced microglial activation. Mediators Inflamm. 2009, 705379. 10.1155/2009/705379 19343193 PMC2662525

[B6] Chen J. Martindale J. L. Cramer C. Gorospe M. Atasoy U. Drew P. D. (2017). The RNA-binding protein HuR contributes to neuroinflammation by promoting C-C chemokine receptor 6 (CCR6) expression on Th17 cells. J. Biol. Chem. 292 (35), 14532–14543. 10.1074/jbc.M117.782771 28684423 PMC5582845

[B5] Chen Y. Jiang T. Chen P. Ouyang J. Xu G. Zeng Z. (2011). Emerging tendency towards autoimmune process in major depressive patients: a novel insight from Th17 cells. Psychiatry Res. 188 (2), 224–230. 10.1016/j.psychres.2010.10.029 21129782

[B7] Cohen H. Ziv Y. Cardon M. Kaplan Z. Matar M. A. Gidron Y. (2006). Maladaptation to mental stress mitigated by the adaptive immune system via depletion of naturally occurring regulatory CD4^+^ CD25^+^ cells. J. Neurobiol. 66 (6), 552–563. 10.1002/neu.20249 16555237

[B8] Corriger A. Pickering G. (2019). Ketamine and depression: a narrative review. Drug Des. Devel. Ther. 13, 3051–3067. 10.2147/DDDT.S221437 PMC6717708 31695324

[B9] Cui Y. Yang Y. Ni Z. Dong Y. Cai G. Foncelle A. (2018). Astroglial Kir4.1 in the lateral habenula drives neuronal bursts in depression. Nature 554 (7692), 323–327. 10.1038/nature25752 29446379

[B10] Davami M. H. Baharlou R. Ahmadi Vasmehjani A. Ghanizadeh A. Keshtkar M. Dezhkam I. (2016). Elevated IL-17 and TGF-β serum levels: a positive correlation between T-helper 17 cell-related pro-inflammatory responses with major depressive disorder. Basic Clin. Neurosci. 7 (2), 137–142. 10.15412/J.BCN.03070207 27303608 PMC4892318

[B11] Davis F. P. Kanno Y. O’Shea J. J. (2015). A metabolic switch for Th17 pathogenicity. Cell 163 (6), 1308–1310. 10.1016/j.cell.2015.11.033 26638065

[B12] Derecki N. C. Cardani A. N. Yang C. H. Quinnies K. M. Crihfield A. Lynch K. R. (2010). Regulation of learning and memory by meningeal immunity: a key role for IL-4. J. Exp. Med. 207 (5), 1067–1080. 10.1084/jem.20091419 20439540 PMC2867291

[B13] Diller M. L. Kudchadkar R. R. Delman K. A. Lawson D. H. Ford M. L. (2016). Balancing inflammation: the link between Th17 and regulatory T cells. Mediators Inflamm., 2016, 6309219. 10.1155/2016/6309219 27413254 PMC4930807

[B14] Fan G. Y. Cherng J. H. Chang S. J. Poongodi R. Chang A. Wu S. T. (2017). The immunomodulatory imbalance in patients with ketamine cystitis. Biomed. Res. Int. 2017, 2329868. 10.1155/2017/2329868 29204439 PMC5674484

[B15] Frenkel D. Huang Z. Maron R. Koldzic D. N. Moskowitz M. A. Weiner H. L. (2005). Neuroprotection by IL-10-producing MOG CD4^+^ T cells following ischemic stroke. J. Neurol. Sci. 233 (1-2), 125–132. 10.1016/j.jns.2005.03.022 15894335

[B16] Freudenberg F. Celikel T. Reif A. (2015). The role of α-amino-3-hydroxy-5-methyl-4-isoxazolepropionic acid (AMPA) receptors in depression: central mediators of pathophysiology and antidepressant activity? Neurosci. Biobehav Rev. 52, 193–206. 10.1016/j.neubiorev.2015.03.005 25783220

[B17] Fung T. C. Olson C. A. Hsiao E. Y. (2017). Interactions between the microbiota, immune and nervous systems in health and disease. Nat. Neurosci. 20 (2), 145–155. 10.1038/nn.4476 28092661 PMC6960010

[B18] Gaffen S. L. (2009). Structure and signalling in the IL-17 receptor family. Nat. Rev. Immunol. 9 (8), 556–567. 10.1038/nri2586 19575028 PMC2821718

[B19] Ghosh R. Kumar P. K. Mitra P. Purohit P. Nebhinani N. Sharma P. (2020). Circulating T helper 17 and IFN-γ positive Th17 cells in major depressive disorder. Behav. Brain Res. 394, 112811. 10.1016/j.bbr.2020.112811 32702351

[B20] Griffiths C. E. M. Fava M. Miller A. H. Russell J. Ball S. G. Xu W. (2017). Impact of ixekizumab treatment on depressive symptoms and systemic inflammation in patients with moderate-to-severe psoriasis: an integrated analysis of three phase 3 clinical studies. Psychother. Psychosom. 86 (5), 260–267. 10.1159/000479163 28903122

[B21] Grosse L. Hoogenboezem T. Ambrée O. Bellingrath S. Jörgens S. de Wit H. J. (2016). Deficiencies of the T and natural killer cell system in major depressive disorder: T regulatory cell defects are associated with inflammatory monocyte activation. Brain Behav. Immun. 54, 38–44. 10.1016/j.bbi.2015.12.003 26674997

[B22] Ho M. F. Zhang C. Zhang L. Li H. Weinshilboum R. M. (2019). Ketamine and active ketamine metabolites regulate STAT3 and the type I interferon pathway in human microglia: molecular mechanisms linked to the antidepressant effects of ketamine. Front. Pharmacol. 10, 1302. 10.3389/fphar.2019.01302 31827434 PMC6848891

[B23] Hong M. Zheng J. Ding Z. Y. Chen J. H. Yu L. Niu Y. (2013). Imbalance between Th17 and Treg cells may play an important role in the development of chronic unpredictable mild stress-induced depression in mice. Neuroimmunomodulation 20 (1), 39–50. 10.1159/000343100 23172104

[B24] Huppert J. Closhen D. Croxford A. White R. Kulig P. Pietrowski E. (2010). Cellular mechanisms of IL-17-induced blood-brain barrier disruption. FASEB J. 24 (4), 1023–1034. 10.1096/fj.09-141978 19940258

[B25] Ichiyama K. Gonzalez-Martin A. Kim B. S. Jin H. Y. Jin W. Xu W. (2016). The MicroRNA-183-96-182 cluster promotes T helper 17 cell pathogenicity by negatively regulating transcription factor Foxo1 expression. Immunity 44 (6), 1284–1298. 10.1016/j.immuni.2016.05.015 27332731 PMC4918454

[B26] Ide S. Ikekubo Y. Mishina M. Hashimoto K. Ikeda K. (2019). Cognitive impairment that is induced by (R)-Ketamine is abolished in NMDA GluN2D receptor subunit knockout mice. Int. J. Neuropsychopharmacol. 22 (7), 449–452. 10.1093/ijnp/pyz025 31135879 PMC6600477

[B27] Kawanokuchi J. Shimizu K. Nitta A. Yamada K. Mizuno T. Takeuchi H. (2008). Production and functions of IL-17 in microglia. J. Neuroimmunol. 194 (1-2), 54–61. 10.1016/j.jneuroim.2007.11.006 18164424

[B28] Ke X. Ding Y. Xu K. He H. Wang D. Deng X. (2018). The profile of cognitive impairments in chronic ketamine users. Psychiatry Res. 266, 124–131. 10.1016/j.psychres.2018.05.050 29864611

[B29] Kim J. W. Kim Y. K. Hwang J. A. Yoon H. K. Ko Y. H. Han C. (2013). Plasma levels of IL-23 and IL-17 before and after antidepressant treatment in patients with major depressive disorder. Psychiatry Investig. 10 (3), 294–299. 10.4306/pi.2013.10.3.294 PMC3843023 24302954

[B31] Kipnis J. Cohen H. Cardon M. Ziv Y. Schwartz M. (2004). T cell deficiency leads to cognitive dysfunction: implications for therapeutic vaccination for schizophrenia and other psychiatric conditions. Proc. Natl. Acad. Sci. USA 101 (21), 8180–8185. 10.1073/pnas.0402268101 15141078 PMC419577

[B32] Kipnis J. Gadani S. Derecki N. C. (2012). Pro-cognitive properties of T cells. Nat. Rev. Immunol. 12 (9), 663–669. 10.1038/nri3280 22903149 PMC4032225

[B30] Kipnis J. Mizrahi T. Yoles E. Ben-Nun A. Schwartz M. Ben-Nur A. (2002). Myelin specific Th1 cells are necessary for post-traumatic protective autoimmunity. J. Neuroimmunol 130 (1-2), 78–85. 10.1016/s0165-5728(02)00219-9 12225890

[B33] Kluger M. A. Meyer M. C. Nosko A. Goerke B. Luig M. Wegscheid C. (2016). RORγt(+)Foxp3(+) cells are an independent bifunctional regulatory T cell lineage and mediate crescentic GN. J. Am. Soc. Nephrol. 27 (2), 454–465. 10.1681/ASN.2014090880 26054541 PMC4731106

[B34] Kolls J. K. Lindén A. (2004). Interleukin-17 family members and inflammation. Immunity 21 (4), 467–476. 10.1016/j.immuni.2004.08.018 15485625

[B35] Korn T. Bettelli E. Oukka M. Kuchroo V. K. (2009). IL-17 and Th17 cells. Annu. Rev. Immunol. 27, 485–517. 10.1146/annurev.immunol.021908.132710 19132915

[B36] Kostic M. Zivkovic N. Cvetanovic A. Stojanovic I. Colic M. (2017). IL-17 signalling in astrocytes promotes glutamate excitotoxicity: indications for the link between inflammatory and neurodegenerative events in multiple sclerosis. Mult. Scler. Relat. Disord. 11, 12–17. 10.1016/j.msard.2016.11.006 28104249

[B37] Krausgruber T. Schiering C. Adelmann K. Harrison O. J. Chomka A. Pearson C. (2016). T-bet is a key modulator of IL-23-driven pathogenic CD4(+) T cell responses in the intestine. Nat. Commun. 7, 11627. 10.1038/ncomms11627 27193261 PMC4874038

[B39] Lee J. E. Lee J. M. Park Y. J. Kim B. S. Jeon Y. T. Chung Y. (2017). Inhibition of autoimmune Th17 cell responses by pain killer ketamine. Oncotarget 8 (52), 89475–89485. 10.18632/oncotarget.18324 29163764 PMC5685685

[B38] Lee Y. Awasthi A. Yosef N. Quintana F. J. Xiao S. Peters A. (2012). Induction and molecular signature of pathogenic TH17 cells. Nat. Immunol. 13 (10), 991–999. 10.1038/ni.2416 22961052 PMC3459594

[B40] Lewitus G. M. Schwartz M. (2009). Behavioral immunization: immunity to self-antigens contributes to psychological stress resilience. Mol. Psychiatry 14 (5), 532–536. 10.1038/mp.2008.103 18779818

[B42] Liu Q. Xin W. He P. Turner D. Yin J. Gan Y. (2014). Interleukin-17 inhibits adult hippocampal neurogenesis. Sci. Rep. 4, 7554. 10.1038/srep07554 25523081 PMC4271266

[B41] Liu Y. Ho R. C. Mak A. (2012). The role of interleukin (IL)-17 in anxiety and depression of patients with rheumatoid arthritis. Int. J. Rheum. Dis. 15 (2), 183–187. 10.1111/j.1756-185X.2011.01673.x 22462422

[B43] McGeachy M. J. Cua D. J. (2008). Th17 cell differentiation: the long and winding road. Immunity 28 (4), 445–453. 10.1016/j.immuni.2008.03.001 18400187

[B44] Moalem G. Leibowitz-Amit R. Yoles E. Mor F. Cohen I. R. Schwartz M. (1999). Autoimmune T cells protect neurons from secondary degeneration after central nervous system axotomy. Nat. Med. 5 (1), 49–55. 10.1038/4734 9883839

[B45] Moynes D. M. Vanner S. J. Lomax A. E. (2014). Participation of interleukin 17A in neuroimmune interactions. Brain Behav. Immun. 41, 1–9. 10.1016/j.bbi.2014.03.004 24642072

[B46] Murrough J. W. Abdallah C. G. Mathew S. J. (2017). Targeting glutamate signalling in depression: progress and prospects. Nat. Rev. Drug Discov. 16 (7), 472–486. 10.1038/nrd.2017.16 28303025

[B47] Nadeem A. Ahmad S. F. Al-Harbi N. O. Fardan A. S. El-Sherbeeny A. M. Ibrahim K. E. (2017). IL-17A causes depression-like symptoms via NFκB and p38MAPK signaling pathways in mice: implications for psoriasis associated depression. Cytokine 97, 14–24. 10.1016/j.cyto.2017.05.018 28570931

[B48] Niebling J. E Rünker A. Schallenberg S. Kretschmer K. Kempermann G. (2014). Myelin-specific T helper 17 cells promote adult hippocampal neurogenesis through indirect mechanisms. F1000Res 3, 169. 10.12688/f1000research.4439.1 25383186 PMC4215755

[B49] Park B. V. Pan F. (2015). The role of nuclear receptors in regulation of Th17/Treg biology and its implications for diseases. Cell Mol. Immunol. 12 (5), 533–542. 10.1038/cmi.2015.21 25958843 PMC4579653

[B50] Poletti S. de Wit H. Mazza E. Wijkhuijs A. J. M. Locatelli C. Aggio V. (2017). Th17 cells correlate positively to the structural and functional integrity of the brain in bipolar depression and healthy controls. Brain Behav. Immun. 61, 317–325. 10.1016/j.bbi.2016.12.020 28025071

[B51] Powell N. MacDonald T. T. (2017). Recent advances in gut immunology. Parasite Immunol. 39 (6). 10.1111/pim.12430 28370104

[B52] Revu S. Wu J. Henkel M. Rittenhouse N. Menk A. Delgoffe G. M. (2018). IL-23 and IL-1β drive human Th17 cell differentiation and metabolic reprogramming in absence of CD28 costimulation. Cell Rep. 22 (10), 2642–2653. 10.1016/j.celrep.2018.02.044 29514093 PMC5884137

[B53] Saraykar S. Cao B. Barroso L. S. Pereira K. S. Bertola L. Nicolau M. (2018). Plasma IL-17A levels in patients with late-life depression. Braz. J. Psychiatry 40 (2), 212–215. 10.1590/1516-4446-2017-2299 29069253 PMC6900762

[B54] Serpe C. J. Coers S. Sanders V. M. Jones K. J. (2003). CD4^+^ T, but not CD8^+^ or B, lymphocytes mediate facial motoneuron survival after facial nerve transection. Brain Behav. Immun. 17 (5), 393–402. 10.1016/s0889-1591(03)00028-x 12946661

[B55] Shen H. Goodall J. C. Hill Gaston J. S. (2009). Frequency and phenotype of peripheral blood Th17 cells in ankylosing spondylitis and rheumatoid arthritis. Arthritis Rheum. 60 (6), 1647–1656. 10.1002/art.24568 19479869

[B56] Sie C. Korn T. Mitsdoerffer M. (2014). Th17 cells in central nervous system autoimmunity. Exp. Neurol. 262 (Pt A), 18–27. 10.1016/j.expneurol.2014.03.009 24681001

[B57] Spanemberg L. Caldieraro M. A. Vares E. A. Wollenhaupt-Aguiar B. Kauer-Sant’Anna M. Kawamoto S. Y. (2014). Biological differences between melancholic and nonmelancholic depression subtyped by the CORE measure. Neuropsychiatr. Dis. Treat. 10, 1523–1531. 10.2147/NDT.S66504 25187716 PMC4149384

[B58] Sun C. Zhang J. Chen L. Liu T. Xu G. Li C. (2017). IL-17 contributed to the neuropathic pain following peripheral nerve injury by promoting astrocyte proliferation and secretion of proinflammatory cytokines. Mol. Med. Rep. 15 (1), 89–96. 10.3892/mmr.2016.6018 27959414 PMC5355678

[B59] Swardfager W. Herrmann N. Andreazza A. C. Swartz R. H. Khan M. M. Black S. E. (2014). Poststroke neuropsychiatric symptoms: relationships with IL-17 and oxidative stress. Biomed. Res. Int. 2014, 245210. 10.1155/2014/245210 25054133 PMC4087285

[B60] Tfilin M. Turgeman G. (2019). Interleukine-17 administration modulates adult hippocampal neurogenesis and improves spatial learning in mice. J. Mol. Neurosci. 69 (2), 254–263. 10.1007/s12031-019-01354-4 31254254

[B61] Waisman A. Hauptmann J. Regen T. (2015). The role of IL-17 in CNS diseases. Acta Neuropathol. 129 (5), 625–637. 10.1007/s00401-015-1402-7 25716179

[B62] Wolf S. A. Steiner B. Akpinarli A. Kammertoens T. Nassenstein C. Braun A. (2009). CD4-positive T lymphocytes provide a neuroimmunological link in the control of adult hippocampal neurogenesis. J. Immunol. 182 (7), 3979–3984. 10.4049/jimmunol.0801218 19299695

[B63] Wolf S. A. Steiner B. Wengner A. Lipp M. Kammertoens T. Kempermann G. (2009). Adaptive peripheral immune response increases proliferation of neural precursor cells in the adult hippocampus. FASEB J. 23 (9), 3121–3128. 10.1096/fj.08-113944 19433626

[B64] Yang J. Sundrud M. S. Skepner J. Yamagata T. (2014). Targeting Th17 cells in autoimmune diseases. Trends Pharmacol. Sci. 35 (10), 493–500. 10.1016/j.tips.2014.07.006 25131183

[B65] Yang Y. Cui Y. Sang K. Dong Y. Ni Z. Ma S. (2018). Ketamine blocks bursting in the lateral habenula to rapidly relieve depression. Nature 554 (7692), 317–322. 10.1038/nature25509 29446381

[B66] Yang Y. Song Y. Zhang X. Zhao W. Ma T. Liu Y. (2020). Ketamine relieves depression-like behaviors induced by chronic postsurgical pain in rats through anti-inflammatory, anti-oxidant effects and regulating BDNF expression. Psychopharmacol. 237 (6), 1657–1669. 10.1007/s00213-020-05490-3 32125485

[B67] Yasuda K. Takeuchi Y. Hirota K. (2019). The pathogenicity of Th17 cells in autoimmune diseases. Semin. Immunopathol. 41 (3), 283–297. 10.1007/s00281-019-00733-8 30891627

[B68] Zanos P. Moaddel R. Morris P. J. Riggs L. M. Highland J. N. Georgiou P. (2018). Ketamine and ketamine metabolite Pharmacology: insights into therapeutic mechanisms. Pharmacol. Rev. 70 (3), 621–660. 10.1124/pr.117.015198 29945898 PMC6020109

[B69] Zimmermann J. Krauthausen M. Hofer M. J. Heneka M. T. Campbell I. L. Müller M. (2013). CNS-targeted production of IL-17A induces glial activation, microvascular pathology and enhances the neuroinflammatory response to systemic endotoxemia. PLoS One 8 (2), e57307. 10.1371/journal.pone.0057307 23468966 PMC3584143

[B71] Ziv Y. Ron N. Butovsky O. Landa G. Sudai E. Greenberg N. (2006). Immune cells contribute to the maintenance of neurogenesis and spatial learning abilities in adulthood. Nat. Neurosci. 9 (2), 268–275. 10.1038/nn1629 16415867

[B70] Ziv Y. Schwartz M. (2008). Orchestrating brain-cell renewal: the role of immune cells in adult neurogenesis in health and disease. Trends Mol. Med. 14 (11), 471–478. 10.1016/j.molmed.2008.09.004 18929506

